# Integration of molecular biology tools for identifying promoters and genes abundantly expressed in flowers of *Oncidium *Gower Ramsey

**DOI:** 10.1186/1471-2229-11-60

**Published:** 2011-04-07

**Authors:** Chen-Tran Hsu, De-Chih Liao, Fu-Hui Wu, Nien-Tze Liu, Shu-Chen Shen, Shu-Jen Chou, Shu-Yun Tung, Chang-Hsien Yang, Ming-Tsair Chan, Choun-Sea Lin

**Affiliations:** 1 Agricultural Biotechnology Research Center, Academia Sinica, Taipei, Taiwan; 2 Scientific Instrument Center, Academia Sinica, Taipei, Taiwan; 3 Institute of Plant and Microbial Biology, Academia Sinica, Taipei, Taiwan; 4 Institute of Molecular Biology, Academia Sinica, Taipei, Taiwan; 5 Institute of Biotechnology, National Chung Hsing University, Taichung, Taiwan; 6 Academia Sinica Biotechnology Center in Southern Taiwan, Tainan, Taiwan

## Abstract

**Background:**

Orchids comprise one of the largest families of flowering plants and generate commercially important flowers. However, model plants, such as *Arabidopsis thaliana *do not contain all plant genes, and agronomic and horticulturally important genera and species must be individually studied.

**Results:**

Several molecular biology tools were used to isolate flower-specific gene promoters from *Oncidium *'Gower Ramsey' (*Onc*. GR). A cDNA library of reproductive tissues was used to construct a microarray in order to compare gene expression in flowers and leaves. Five genes were highly expressed in flower tissues, and the subcellular locations of the corresponding proteins were identified using lip transient transformation with fluorescent protein-fusion constructs. BAC clones of the 5 genes, together with 7 previously published flower- and reproductive growth-specific genes in *Onc*. GR, were identified for cloning of their promoter regions. Interestingly, 3 of the 5 novel flower-abundant genes were putative trypsin inhibitor (*TI*) genes (*OnTI1*, *OnTI2 *and *OnTI3*), which were tandemly duplicated in the same BAC clone. Their promoters were identified using transient GUS reporter gene transformation and stable *A. thaliana *transformation analyses.

**Conclusions:**

By combining cDNA microarray, BAC library, and bombardment assay techniques, we successfully identified flower-directed orchid genes and promoters.

## Background

The Orchidaceae family comprises an estimated 35,000 species and is one of the largest families of flowering plants. The Oncidiinae subtribe consists of ~70 closely related genera and >1400 species, of which *Oncidium *is the largest genus [1,2]. Like other orchids, Oncidiinae can be easily crossed intergenerically, or across species, to produce flowers with unique colors, fragrances and shapes. *Oncidium *has become a commercially important flower in the orchid industry. *Oncidium *'Gower Ramsey' (*Onc*. GR) is one of the most important *Oncidium *cut-flower varieties; it is an interspecific hybrid derived from *Onc. flexuosum*, *Onc. sphacelatum *and *Onc. varicosum*. *Onc*. GR is a yellow flower variety that can flower year-round. The length of inflorescence is ~1 m, with hundreds of ca. 4 cm flowers.

Functional genomic studies of orchids remain a challenge owing to large genome size, low transformation efficiency and long life cycles [3]. However, gene transformation of *Onc*. GR has been established [4], offering an alternative strategy for *Oncidium *breeding and making it a priority to investigate and obtain *Oncidium *promoters. To date, several strategies have been used to investigate orchids at the genomic level. Sequence homology searches have identified homologous genes in *Oncidium *[5-11], and expressed sequence tag (EST) databases have been used for gene cloning [12-18]. Because model plants, such as rice and *A. thaliana*, do not contain all plant genes, and because some genes related to the unique morphological and physiological characteristics of *Oncidium*, such as the flower and pseudobulbs cannot be identified using sequence homology, an *Oncidium*-specific cDNA library of pseudobulbs and flowers has been established that contains a large amount of genetic information [12-18]. However, gene expression patterns cannot be predicted by nucleic acid sequences. Furthermore, several of the non-model plant EST sequences are not full-length sequences.

To clone full-length genes and promoters, further processing is necessary, such as rapid amplification of complementary DNA ends (RACE) for full-length cDNA, or genomic walking for promoter studies [8,15,16]. These techniques are difficult to apply to *Onc*. GR because its genome is complex and has not been sequenced. Bacterial artificial chromosome (BAC) libraries are an alternative tool for full-length gene and promoter cloning. To obtain such libraries, genomic DNA is cut into pieces of ~100 kb, cloned into a vector and stored in bacteria, making it is easier to obtain the promoter and the full length of the target gene without interference from homologs in the genome. Various strategies can then be used to identify the clones that contain target genes [19-22], and the identified clones can be sequenced directly to obtain the full-length gene sequence.

In this report, a cDNA microarray, a BAC library and a bombardment assay were combined to establish a novel platform that was used to identify and clone the *Onc*. GR genes and promoters abundantly expressed in *Onc*. GR flowers. This approach, combining multiple tools provides a fast, easy to use and convenient strategy for obtaining useful genetic information about *Oncidium*.

## Results

### Using cDNA microarray to identify genes highly expressed in flowers

A cDNA microarray was used to identify genes that are abundantly expressed in flowers. PCR products of 1065 clones from the cDNA library of *Onc*. GR were spotted on to slides to establish a flower-derived microarray. A total of 77 clones were upregulated by >3-fold and 42 clones were downregulated >3-fold relative to the leaves (data not shown).

Sequencing revealed that several clones were repeated. Among the 77 clones corresponding to genes highly expressed in flowers, 57 were unique genes. Among the clones corresponding to genes highly expressed in leaves, 3 were related to photosynthesis/chloroplasts (chloroplast chlorophyll a/b-binding protein, NADH dehydrogenase, and photosystem II 10 kDa protein) as expected; photosynthesis-related genes were highly expressed in leaves.

**Figure 1:**
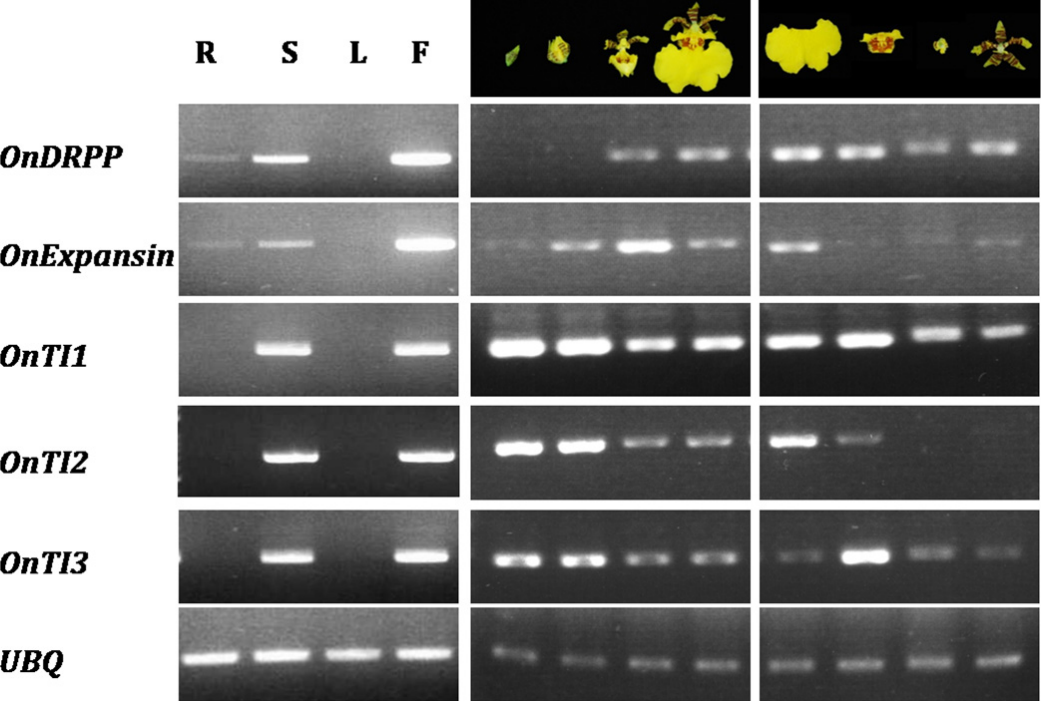
**RT-PCR confirmed that genes identified by microarray were highly but variably expressed in reproductive organs according to the developmental stage and tissue**. Total RNA was isolated from various organs (R, root; S, stalk; L, leaf; F, flower) during different developmental stages (green bud, showing color, expanding, full bloom), and from various parts of the flower (lip, callus, reproductive column, and sepal and petal). The genes included *Oncidium Expansin *(*OnExpansin*), *Oncidium Disease Resistant Response Protein *(*OnDRRP*) and *Oncidium Trypsin inhibitor *(*OnTI1*, *OnTI2*, and *OnTI3*). Each experiment was carried out in triplicate. Ubiquitin was used to measure the amount of RNA used for each RT-PCR reaction.

**Figure 2:**
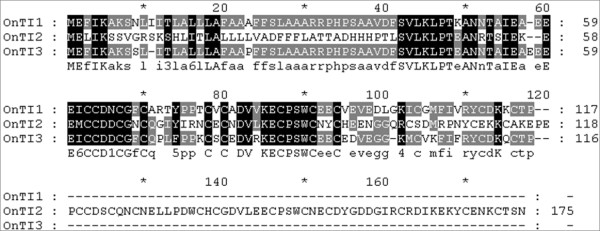
**Alignment of amino acid sequences of OnTI1, OnTI2 and OnTI3**. Comparison of the cDNA amino acid sequences of OnTI1, OnTI2 and OnTI3. Amino acids identical in all the proteins are presented in *black*; those conserved in at least 2 sequences are *shaded*.

Genes in which the flower/leaf expression ratio was >7.5 are presented in Table 1. *Gastrodianin *and *Aquaporin *were duplicated in the microarray but appeared as different ratios. As no suitable RT-PCR primers for the gene similar to CAE01572.2 could be identified, RT-PCR of the remaining 6 genes was performed to validate the microarray results. *Cytosolic malate dehydrogenase *was the only gene whose RT-PCR results were inconsistent with the microarray. The other 5 genes were highly expressed in reproductive tissues including flowers and stalks (Figure 1). Three of them, *OnTI1*, *OnTI2*, and *OnTI3*, shared sequence homology with known trypsin inhibitors (TI, Figure 2) and probably have similar functions. The remaining two, although highly expressed in flowers, were expressed at different development stages or in different flower organs (Figure 1). *Disease resistance response protein *(*OnDRRP*) was expressed in fully blooming flowers and *Expansin *(*OnExpansin*) was highly expressed in the lip (labellum) extending stage. The 3 trypsin inhibitor genes were expressed at all stages, but most abundantly during the flower bud stage. In reproductive organs, *OnExpansin *and *OnTI2 *were predominantly expressed in the lips. *OnTI3 *was highly expressed in the callus.

**Table 1:** *Onc. *Gower Ramsey genes that are abundantly expressed (> 7.5×) or repressed (< 0.06×) in flower tissues

Putative function	Clone ID	GenBank No.	F/L
**Flower abundant**			
*OnDRRP*	S1H08	HS524704	22.86+9.50
*Cytosolic malate dehydrogenase*	08H08	HS522502	16.81+10.64
*OnExpansin*	02C02	HS521943	14.59+8.26
*OnTI3*	10A09	HS522609	10.85+4.89
*CAE01572.2_like*	06A05	HS522251	10.17+4.44
*Gastrodianin-1*	S1G11	HS524695	8.82+4.51
*Gastrodianin-2*	S1E09	HS524669	8.77+4.97
*Aquaporin*	07D11	HS522379	8.08+4.30
*OnTI1*	03G05	HS522068	8.07+4.76
*OnTI2*	S1D01	HS524649	7.64+1.08
**Flower repression**			
*3-phosphoinositide-dependent protein kinase*	03D08	HS522037	0.01+0.00
*Metallothionein*	07D07	HS522375	0.01+0.00
*NP_085475.1 like*	09G06	HS522583	0.02+0.01
*NADH dehydrogenase subunit*	06F02	HS522306	0.02+0.01
*OnHy_06B11*	06B11	HS522268	0.02+0.02
*Chlorophyll a/b-binding protein*	S1D02	HS524650	0.03+0.02
*40S ribosomal protein*	06D01	HS522282	0.05+0.04
*OnHy_S1A10*	S1A10	HS524622	0.06+0.03

Values are presented as average ± SD of 3 biological replicates (*n *= 3). "*OnHy*" denotes that no similar protein was identified using BlastX.

### Promoter cloning using a BAC library

**Figure 3:**
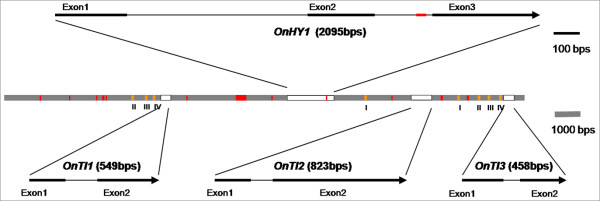
**Gene Structure of *OnTI***. Genes are marked by white boxes. Intergene spaces are denoted by a gray line. Introns are denoted by thin lines. The lengths of the exons, genes and intergene space (in base pairs) are indicated. Red, tandem repeat; orange, conserved regions in the *OnTI *promoters.

Having used RT-PCR to confirm that these 5 genes were highly expressed in flowers, they were used for further promoter studies. BAC clones that contained the target genes were used for promoter cloning. There are ~140,000 clones in the *Onc*. GR BAC library. Because the target gene sequences were known, PCR was used for screening. BAC screening was performed on a total of 12 genes; the 5 genes highly expressed in flowers as detailed above, and 7 previously published *Oncidium *flower-related genes (Table 2). These 12 genes were located in 10 different clones. Interestingly, the 3 trypsin inhibitor genes were located in the same clone, and tandemly duplicated sequences were found in *OnTI2 *and *OnTI3*. A hypothetical gene, *OnHY1*, was located between *OnTI1 *and *OnTI2 *(Figure 3). The putative protein sequence contains a Bowman-Birk serine protease inhibitor domain in the N-terminal region, similar to *Lens culinaris *trypsin inhibitor [GenBank: CAH04446.1]; and an amino acid sequence between 150 aa and 200 aa that is similar to a transposase domain.

**Table 2:** Primers used for RT-PCR and BAC screening

Gene	Forward primer	Reverse primer	Clone ID	GenBank No.
UBQ	ACA TTC AGA AGG AGT CAA CCC	CGATGTCGATTTCGATTTCC		
*OnDRRP*	TGAAAAAGAAACCCATCTGCA	GCCCATAGGTGCCAATATTT	P-5-O-22	HQ832781
*OnExpansin*	ACGCAACTTTCTATGGCGG	AAGCAACCACAGCTCCAAGT	O-1-O-24	HQ832782
*OnTI1*	ATCACTTTGGCTCTGCTGCTT	TGCCGAGGTCCTCGACTTCCA	J-1-K-16	HQ832783
*OnTI2*	AAGAAGAACTCCCCACAAGAA	AGGTTGATCGATCGAAGCA	J-1-K-16	HQ832783
*OnTI3*	ATCACTTTGGCTCTGCTGCTT	AGCAATGAATGACGATCGAC	J-1-K-16	HQ832783
*OMADS3*	GAGGTATCAGCAAGTTACCG	CGAACGATCTTAATCGACTC	45-3-B-1	HQ832787
*OMADS6*	AAACCCAGAGTAGTCAGCAG	GTCATATCCCATTGCATGA	73-1-K-8	HQ832788
*OMADS8*	ATGGAAGGCAGCATGAGAGAAC	AAAGCGTTAGCATTGTTACTTGTTT	AAP-1-C-19	HQ832789
*OMADS9*	GATAAACCAAAACCTGAGGA	TTTTGTAGGTATCGGTCTGG	L-1-P-13	HQ832790
*OnFT*	ATTGTAGGACGAGTGATTGG	TACTTGGACTTGGAGCATCT	Q-1-I-4	HQ832784
*OnLeafy*	TTCCTGGATCTCAACATCAT	TGCTGAAATCCTCAAACTTCA	Orp-2-F-21	HQ832785
*OnTFL*	TTGTAGTTGGTAGAGTTATAGGAGAAG	ATCAGTCATAATCAGTGTGAAGAAAG	Q-1-B-10	HQ832786

### Identifying protein sub-cellular localization using fusion with fluorescent proteins

**Figure 4:**
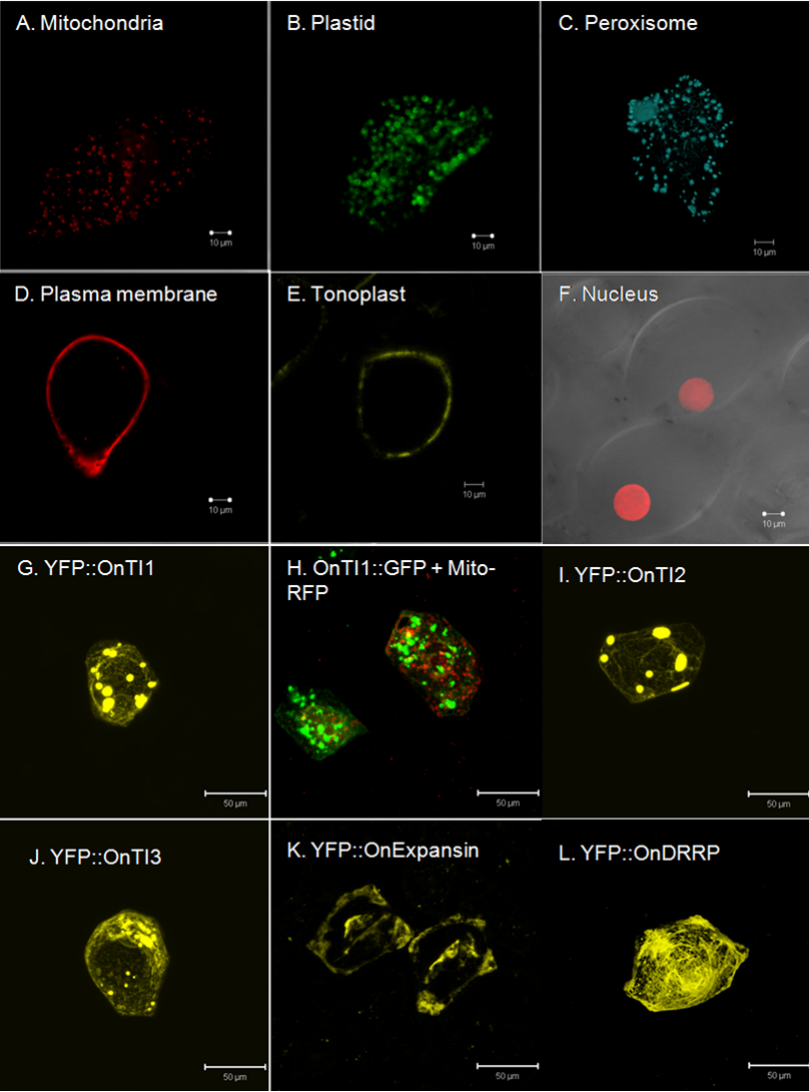
**Characteristic features of organelle markers and subcellular location of proteins of flower-abundant genes in *Onc*. Gower Ramsey**. **A**. Mitochondrial marker: the first 29 amino acids of yeast cytochrome c oxidase IV fused with RFP. **B**. Plastid marker: the targeting sequence (first 79 aa) of the small subunit of tobacco rubisco fused with GFP. **C**. CFP peroxisome marker: cytoplasmic tail and transmembrane domain of soybean 1, 2-mannosidase I fused with CFP. **D**. RFP plasma membrane marker: the full length of AtPIP2A, a plasma membrane aquaporin fused with RFP. **E**. YFP vacuole marker: γ-TIP, an aquaporin of the vacuolar membrane fused with YFP. **F**. Nuclear marker: NLS domain of VirD2 fused with mCherry. **G**. YFP: OnTI1: YFP fused with the N-terminus of OnTI1 protein. **H**. OnTI1::GFP + Mito-RFP: OnTI1::GFP and Mitochondria RFP marker were co-transformed to the cells. **I**. YFP::OnTI2: YFP fused with the N-terminus of OnTI1 protein. **J**. YFP::OnTI3: YFP fused with the N-terminus of OnTI3 protein. **K**. YFP::OnExpansin: YFP fused with the N-terminus of OnExpansin protein. **L**. YFP::OnDRRP: YFP fused with the N-terminus of OnDRRP protein.

*Oncidium *lip bombardment-mediated transformation was used to investigate the subcellular location of the protein products of the particular genes that were identified by microarray. Published protein markers were used to identify the organelles in the *Oncidium *cells of which the endomembrane system was most difficult to distinguish. Multiple protein markers derived from different plant species [23] indicated that these marker plasmids can be delivered into cells to synthesize fluorescent proteins (Figure 4A-E). Not only could the endomembrane systems be identified, but VirD2-NLS -mCherry (Figure 4F) could be used as a nuclear marker [24].

For the *Oncidium *genes investigated, no difference in the fluorescence patterns was observed when proteins were expressed as N- or C-terminal fusions with a fluorescent protein (Figure 4G and 4H, OnTI1). The 3 OnTI proteins were seen as aggregated particles in the cells (Figure 4G-J). The subcellular locations of these proteins differed from endomembrane markers, such as mitochondria (Figure 4H). For YFP-OnExpasin, fluorescent signals were evident in the intercellular space and at the cell wall (Figure 4K), and for OnDRRP fluorescent signals appeared as a network system throughout the cell (Figure 4L).

### Use of multiple tools to identify promoters

**Figure 5:**
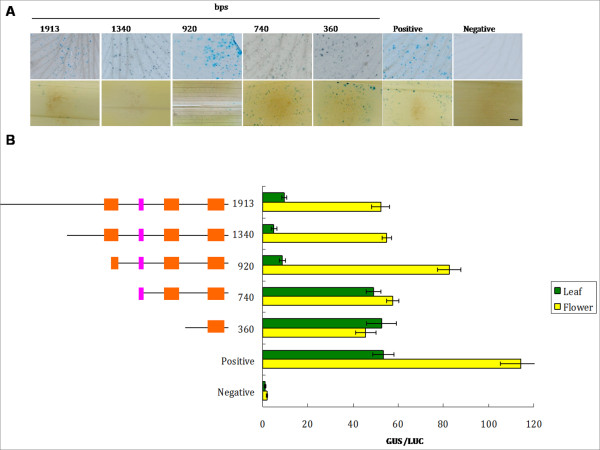
**Promoter study of *OnTI1***. Plasmids harboring various lengths of *OnTI1 *promoter fused with *GUS *were delivered to the lips and leaves of *Oncidium *Gower Ramsey. (A) The transformed tissues are stained to demonstrate GUS activity. The number on at the top is the length of the promoter. (B) Quantitative analysis of GUS activity. Orange boxes, the conserved regions II, III and IV of the *OnTI *promoter region. *Oncidium alcohol acyl-transferase *500 bp promoter-*GUS *was used as the positive control, with the negative control being just the vector.

**Figure 6:**
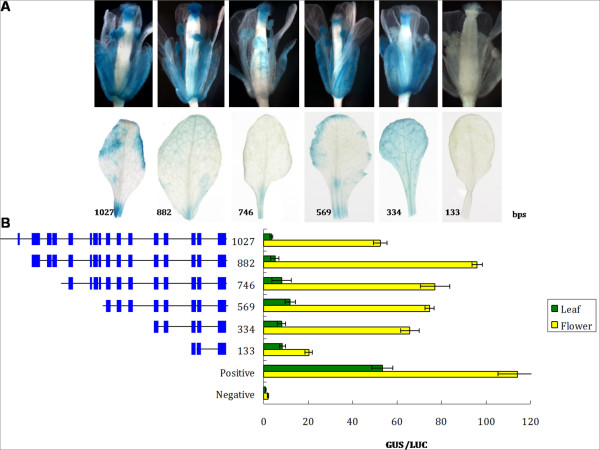
**Promoter study of *OnExpansin***. Plasmids with various lengths of *OnExpansin *promoter fused with *GUS *were transformed into *Arabidopsis thaliana *(A) or delivered to the lips and leaves of *Oncidium *Gower Ramsey. (B). The number indicates the length of the promoter. The blue box denotes the putative floral-related transcription binding site.

**Figure 7:**
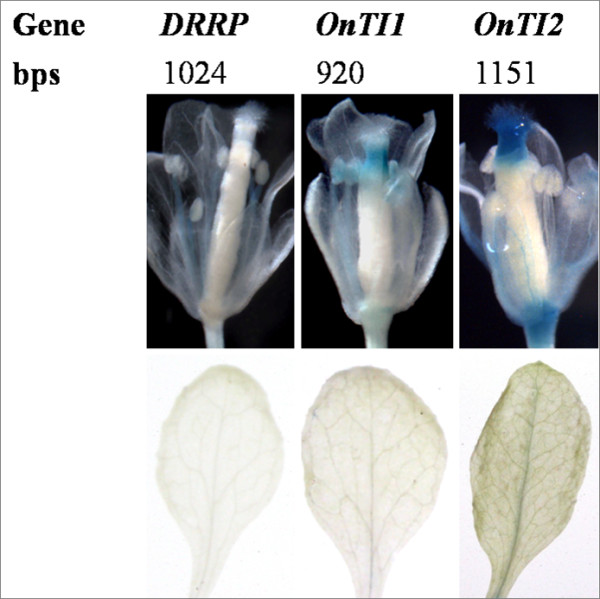
***Oncidium *promoters that are highly expressed in *Oncidium *flowers**. The Oncidium transient transformation study: clones with a high flower/leaf GUS activity ratio were transformed into *Arabidopsis thaliana*. The promoters included *Oncidium Disease Resistant Response Protein *(*OnDRRP*) and *Oncidium Trypsin inhibitor *(*OnTI1 *and *OnTI2*). The number indicates the length of the promoter.

The 5 genes of interest were expressed in the lips; therefore, the *Onc*. GR lip was used for transient transformation. *Oncidium alcohol acyl-transferase *can be expressed in the leaves and flowers; its promoter (500 bp) was used as a positive control to demonstrate successful transformation. To investigate the promoter of *OnTI1*, various lengths (360, 740, 920, 1340, and 1913 bp) of the promoter region fused to the GUS reporter gene were introduced into the cells using the bombardment method. Plasmid pJD301 containing 35S-*LUC *was co-bombarded as a reference control. The highest GUS activity was evident with the 920 bp length promoter. Interestingly, similar GUS activity was detected in the leaves using the leaves using the 360 and 740 bp lengths of the promoter region. GUS activities in the leaves were repressed in the transformants that had a promoter length of equal to or longer than 920 bp (Figure 5). For *OnExpansin*, GUS activity in the leaves of all promoter transformants was low. GUS activity in the flower was correlated with promoter length, except for the 1027 bp region, which had significantly reduced activity (Figure 6). Different lengths of *OnExpansin *promoter-GUS constructs were transformed into *A. thaliana*. With the exception of the 133 bp transformants, GUS activity was detected in flowers and minimal activity was present in the leaves (Figure 6). Various lengths of *OnTI2 *and *OnDRRP *promoters were constructed and a promoter assay was conducted (data not shown). The constructs of *OnExpansin*, *OnT1 *and *OnT2 *yielding the highest flower/leaf GUS activity were then transformed into *A. thaliana*. The transformants of *OnExpansin *had the highest GUS activity in the flowers (Figure 6), whereas that of *OnDRRP *had the lowest (Figure 7). *OnExpansin *had GUS activity in the leaves (Figure 6). The flower GUS activity patterns for both *OnTI1 *and *OnTI2 *promoters were similar. Staining was observed at the top of the styles and at the junction of the pedicel and flowers (Figure 7).

## Discussion

### Identification of *Oncidium *reproductive-specific expression of genes using cDNA microarray

The aim of this study was to establish a successful combination of integrated tools to obtain genetic information about the commercially important cut flower *Onc*. GR. A combination of a cDNA library, a microarray, a BAC library and transient transformation was effective. However, the microarray and cDNA library that was used had several limitations: (1) In gene families that have conserved regions and share sequence identity, binding occurs that can limit the specificity of the data. For example, we found that *gastrodianin*, *aquaporin *and *cytosolic malate dehydrogenase *gave false positives. (2) The clone number was limited. There were only 1065 clones in the microarray, which cover only a fraction of the *Oncidium *genome. The estimated genome size is 1C = 2.84 pg, http://data.kew.org/cvalues/CvalServlet?querytype=1. The estimated coverage of the *Onc*. GR BAC library is thus 1.28 fold, thereby limiting its possible uses. (3) Only a few genes that are highly expressed in leaves were identified because the microarray was composed from a flower cDNA library. To widen the use of this array, more sequence information needs to be integrated. For example, further libraries must be derived from different tissues and treatments. Sequences from next generation sequencing are an alternative resource for obtaining this data. In comparison to the traditionally employed method (*i.e*. construction of an EST library, storage and sequencing of each clone using Sanger sequencing technology), using high-throughput approaches allows several thousand ESTs to be obtained cost-effectively from different tissues with less space and effort. Specific gene sequences can then be printed and a microarray yielding more detailed data can be useful for a variety of applications.

### BAC library construction is a useful tool for cloning promoters

Polyploidy is a common phenomenon in crop species. In the indigenous species of *Oncidium*, the chromosome number is 2*n *= 56 http://data.kew.org/cvalues/CvalServlet?querytype=1; however, the chromosome number in *Onc*. GR is 112. Therefore, it is expected that there are several homologous genes in the genome of *Onc*. GR. In addition, tandem duplication, such as that found in the *OnTI *genes, or tandem repeat sequences such as those found in *OnFT *and *OMADS9*, would render genome walking using a PCR strategy particularly difficult to perform (Table 3). In many cases, it would take several months to identify a single gene. By screening a BAC library, target genes are narrowed down to those with lengths of 100 kb, thereby reducing the problems related to homologous genes, tandem repeat sequences and secondary structure. In addition, the PCR strategy used herein can identify the BAC clone containing a target gene within a week, and regions of interest can be sequenced using BAC End Sequencing (BES).

**Table 3:** Tandem repeats in the promoter and gene sequences used in this report.

Gene	Position	Repeat	Copies
*OnExpansin*	-1478	AATAAA	33
*OnTI1*	-3692	A	34
	-2047	CT	14.5
*OnTI2*	-7766	TTA	167
	-6130	TA	26
*OnTI3*	-2662	TTA	23.7
	-1489	AAT	30
*OMADS3*	-1003	TAT	56.7
*OMADS6*	-1234	ATA	13.3
	1079	A	26
*OMADS9*	-66	CTT	8.7
*OnFT*	-1167	TAA	25
*OnLeafy*	-960	TTA	22.3

Two strategies are used for BAC library screening: hybridization and PCR screening. As the gene sequences of the target genes were known in this study, the PCR screening strategy could be adopted. Recent improvements in PCR technology and protocols have made BAC screening more efficient and several genes have been successfully cloned using PCR to screen BAC libraries [19-22]. We thus used this strategy to obtain BAC clones containing genes of interest in the *Onc*. GR library.

### Three trypsin inhibitor genes, *OnTI1*, *OnTI2 *and *OnTI3*, which are highly expressed in flowers, are tandemly duplicated

Three tandemly duplicated genes, *OnTI1*, *OnTI2 *and *OnTI *that are highly expressed in flowers were identified. Gene duplications that encode similar gene functions are a common phenomenon in plants and are thought to have contributed to the origin of evolutionary 'novelties' [25]. For example, it has been proposed that in the early evolution of orchids, two rounds of *DEFICENS*-like MADS-box gene duplications generated the genes that were probably recruited to distinguish the different types of orchid perianth organs [25]. Information about tandem duplicates can be useful in investigations pertaining to gene duplication. For example, the *cinnamyl alcohol dehydrogenase *gene [26], the *R2R3-MYB *family of transcription factors genes [27] and *NAC *domain transcription factors genes [28] are tandemly duplicated in *Populus trichocarpa*. These genes have been duplicated from the same ancestral gene, allowing the expression pattern of these genes to be correlated. An investigation of the gene locations of the NAC domain transcription factors in *Populus trichocarpa *showed that 6 pairs of NACs are present as tandem duplicates, represented in tandem clusters of 2 or 3 genes each. In the tandemly duplicated clusters with 3 genes, the expression patterns of 2 of the genes were found almost identical. However, in the tandemly duplicated clusters with 2 genes, the gene expression levels differed significantly [28]. In the current study, the expression patterns of *OnTI *genes were similar. On the basis of sequence homology, we discovered 4 conserved regions upstream of *OnTI3 *similar to *OnTI2 *(region 1) and *OnTI1 *(regions 2-4). We tentatively suggest that these *OnTI*s may be derived from the same ancestral gene.

Several di- or tri-nucleotide tandem repeats were evident in the flower-related genes (Table 3). Because information on *Oncidium *is limited, the biological significance of tandem repeats in these genes remains unclear. The end sequencing of this BAC library may provide suitable information for identifying the relationship between flower-related genes and tandem repeat sequences.

### Transient transformation is a suitable tool for determining the subcellular localization of protein

The subcellular location of a protein is related to its function. For example, photosynthesis-related proteins are located in chloroplasts. Therefore, experiments aimed at determining the specific localization of proteins can provide information on biological processes [29]. Computational prediction is one method used to investigate the subcellular localization of a protein [29]. However, as yet, no suitable reference database exists for *Oncidium*. Experimentally, the subcellular localization of a protein can be studied by imaging it after fusion with a fluorescent protein [30,31]. However, no suitable protocol for investigating subcellular localization has so far been established for orchids. In this report, a transient transformation system for the orchid lip using markers derived from different species as fluorescent markers was established to study subcellular localization of proteins.

Trypsin inhibitors can be used to reduce trypsin activity, which can play an active role against pests and diseases [32]. The expression of trypsin inhibitor genes can also be induced by water stress [33] and stress-related plant growth regulators [34,35]. Constitutive expression of a trypsin inhibitor can improve plant tolerance to abiotic stress [34,35]. Trypsin inhibitors are present in all protein bodies, and to a lesser extent in the nucleus and intercellular space [36,37]. Here, we found that OnTI proteins can form particles similar to protein bodies, but they were not in the nucleus or intercellular space.

Expansins are a superfamily of proteins crucial in loosening the cell wall. The expansins consist of 2 domains, the glycoside hydrolase family 45 (GH45) catalytic domain and group-2 grass pollen allergens. Experimental evidence indicates that expansins can induce slippage of cellulose microfibrils in the cell wall which becomes loosened [38]. The expansin was located in the cell wall and in the intercell wall spaces [39,40]. The fluorescent signal for OnExpansin was located around the cell wall; according to the results obtained using RT-PCR, *OnExpansin *was highly expressed in the lips and during lip expansion. Therefore, this gene may be correlated with *Onc*. GR lip development.

In summary, the localizations of the proteins we investigated are correlated with their predicted functions, but the roles of these genes during *Oncidium *flower development are unknown as their overexpression in *A. thaliana *flowers did not result in any significant change in terms of flowering time or morphology.

### Useful genetic information can be mined using this integrated platform

Promoters of *Oncidium *were successfully cloned using a combination of a cDNA library, microarray, BAC library and transient transformation. Transformation of *Oncidium *is time-consuming and requires considerable human resources. Use of a transient expression system reduced the time required to obtain preliminary information to ~1 week. This approach is thus more time-efficient than genomic walking and stable transformation methods, and allows investigators to estimate experimental priorities.

There are 4 conserved regions in the promoter regions of *OnTI *genes. The *OnTI1 *promoter study demonstrated that box 1, box 3 and box 4 were not related to flower expression. The *OnTI2 *promoter, which does not have these regions, can be expressed in flowers. The most important region controlling the repression in leaves is situated between box 2 and the repeat region. There is a potential Agamous binding site in this region and there is a similar region in the *OnTI2 *promoter region (TAATGTTACGAAATAAAATATCACTCCTGAATATA). Unlike the repression of *OnTI2 *in leaves, the most important region for flower expression in *OnExpansin *is located between -113 to -334 bp. It is expected that the regulation of *OnExpansin *expression is different from that of *OnTI2*. Interestingly, 2 potential TF-binding domains (an Agamous and an AtHB9 binding site) are flower or development related. The relevance of the Agamous binding site for gene repression in leaves and flower expression, however, requires further investigation.

The promoter regions of *OnTI*, *OnExpansin *and *Oncidium MADS *genes contain nucleotide tandem repeat sequences (Table 3). However, promoter studies demonstrated that the tandem repeats in *OnTI1 *and *OnExpansin *promoters are not related to gene expression. According to our data, the promoter region controlling flower/leaf expression is within 1 kb of the promoter. Analysis of other gene promoters (*OnTI1 *and *OnDRRP*) produced similar results (data not shown).

The clones which contain ~ 1 kb promoter regions fused with *GUS *were transformed into *A. thaliana*. Although GUS staining was more prominent in flowers, there were some unexpected results. In *OnExpansin*, GUS staining was evident in the leaves despite the RT-PCR results demonstrating that *OnExpansin *is predominantly expressed in the lips of *Oncidium*. In *A. thaliana*, GUS was weakly expressed in petals, but highly expressed in anthers and styles (Figure 6). The *OnTI *genes were predominantly expressed in the *Oncidium *lip and callus. However, there was no GUS staining in the petals of the *A. thaliana *transformants. These results may be due to the absence of a transcription factor that can recognize the *Oncidium *binding site, highlighting the necessity of identifying species-specific promoters. The promoters we found were only 1 kb in size. The region that controls the specific organ of interest may not have been included, producing unexpected results in stable *A. thaliana *transformation.

## Conclusions

A cDNA library, a microarray, a BAC library and transient transformation were combined to identify gene promoters highly expressed in the flowers of *Oncidium *Gower Ramsey, a commercially important cut flower. Classical approaches of identifying orchid genes and promoters - in particular the genome walking method - cannot easily be performed when regions of high DNA sequence homology tandem repeats and tandemly duplicated genes are present. Gene sequences of interest were identified successfully using BAC sequencing. Using lip transient transformation, GUS reporter gene fusion constructs with various lengths of promoters were introduced into the lip to determine promoter activity. Furthermore, the subcellular localization of proteins encoded in these genes was also determined in this system. With this combination of approaches, 5 novel *Oncidium *gene promoters of genes abundantly expressed in flowers were cloned and confirmed. These promoters can be used to express genes in floral organs and change the flower phenotype without affecting the vegetative tissues.

## Methods

### Plant materials

Flowering *Onc*. GR (a tetraploid interspecific hybrid) were obtained from a local grower (Yung Hsin Orchid Nursery, Taichung, Taiwan). The orchids were maintained in the greenhouse at Academia Sinica, Taipei, Taiwan. A voucher specimen was deposited at the National Museum of Natural Science, Taichung, Taiwan.

### *Onc*. Gower Ramsey flower cDNA library construction

*Onc*. GR flowers were used as the materials for cDNA library construction. Total RNA and poly(A)+ mRNA were isolated using Trizol reagent (Invitrogen, Carlsbed, CA, USA) and the Oligotex Midi mRNA kit (Qiagen, Venlo, The Netherlands), respectively, according to the manufacturer's instructions. The cDNA library was constructed using the Long Distance PCR SMART cDNA Library Construction kit (Clontech, Mountain View, CA, USA) following the manufacturer's instructions. The cDNAs were cloned into the pDNR-LIB vector (Clontech). Colonies were picked up, collected in 96-well plates, and stored at -80°C.

### Microarray preparation

Microarray preparation followed the procedure described by Wu et al. [41] for the preparation of a bamboo microarray. A total of 1065 cDNAs [GenBank: HS521830-HS522791; HS524614-HS524707] derived from the *Onc*. GR flower cDNA library were amplified using PCR, incorporating the T3 and M13 reverse universal primers. The PCR products were purified using the MultiScreen PCR_96 _Filter Plate (Millipore Corp., Bedford, MA, USA) and eluted with 100 μl of 0.1× TE buffer (1 mM Tris and 0.1 mM EDTA, pH 8.0). Purified PCR products were printed on GAPS II-coated slides (Corning, New York, NY) using the OmniGrid 100 microarray (Genomic Solutions, Ann Arbor, MI, USA), and arranged into two 1.8 × 1.8-cm arrays (spot size: 100 μm). After printing, the slides were left to dry overnight. These DNAs were cross-linked to the slide by baking the array for 2 h at 80°C.

Total RNA from leaves and flowers (25 μg) was used for cDNA synthesis and labeling with either Cy3 or Cy5 dye, using the 3DNA Expression Array Detection kit for microarrays (Array 50, version 2, Genisphere, Hatfield, PA, USA). cDNA hybridization and washing procedures were performed according to the manufacturer's instructions. All experiments were carried out in 3 biological replicates (*n *= 3). Detailed information of the microarray has been deposited in the NCBI GEO database [GEO: GSE26504].

### Semi-quantification using RT-PCR

Total RNA (5 μg) extracted from various tissues was subjected to RT-PCR. First-strand cDNAs were synthesized using M-MLV reverse transcriptase (RNase H Minus, Point mutant; Promega, Madison, WI, USA) and a poly(dT) primer. Each gene was amplified for 25 cycles using primers specific for each gene. *Onc*. GR ubiquitin was used as an internal control. The primers are given in Table 2.

### BAC library construction

Young *Onc*. GR leaves (200 g) were collected for isolation of high molecular weight DNA according to Zhang et al. [42]. The DNA was sheared randomly, and the fragments ligated into the pSMART-BAC vector (Lucigen, Middleton, WI, USA). The ligated DNA was transfected into *E. coli *strain 10G BAC-Optimized Electrocompetent cells (Lucigen).

### Identification of BAC clones containing target genes using PCR

**Figure 8:**
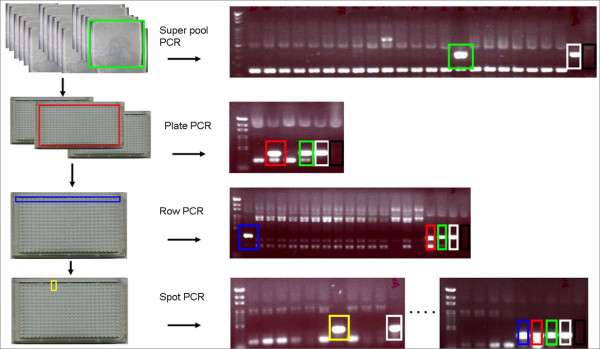
**Strategy for identification of BAC clones containing genes of interest**. Genes of interest or partial *Oncidium *sequences were used for the BAC library screening. Primers were designed and genomic DNA PCR was performed before the screening. The products were sequenced to confirm results. After superpooling, plate PCR, row PCR and spot PCR, BAC clones containing genes of interest were identified. Black box, negative control; white box, genome DNA positive control with genomic DNA; green box, superpool control; red box, plate control; blue box, row control; yellow box, identified clone.

The BAC library of *Onc*. GR DNA in *E. coli *was spread on solid medium plates (23 × 23 cm) containing Luria broth (LB) supplemented with 25 mg/L chloramphenicol. Each plate contained ~1000 clones. After overnight incubation at 37°C, individual clones were picked up by a Q-bot robot (Genetix, New Milton, UK) and placed into a 384-well plate that contained liquid LB medium with 12.5 mg/L chloramphenicol. Clones the robot failed to identify were picked up manually. The 384-well plates were incubated at 37°C overnight and stored at -80°C. The plates were washed with LB liquid medium containing 12.5 mg/L chloramphenicol, incubated overnight at 37°C and stored at -80°C for the superpool (Figure 8).

Sequences derived from the microarray experiments and the published flower-related genes were used to design primers. Primers that could amplify predicted genomic regions in the presence of *Onc*. GR genomic DNA (positive control, white box, Figure 8) were used for further screening. After superpool screening (149 reactions), plate screening (2-5 reactions) was performed, and row (16 reactions) and spot screening (24 reactions) were used to identify clones containing genes of interest.

### BAC plasmid isolation and sequencing

BAC plasmids were isolated using the NucleoBond BAC 100 kit (NucleoSpin blood, Macherey-Nagel GmbH & Co KG, Germany) following the manufacturer's instructions, and sequenced using the Big Dye™ Terminator Cycle Sequencing Ready Reaction kit and an automated sequencer (Perkin-Elmer Applied Biosystems, CA, USA).

### Transient transformation

Bombardment assay was conducted as outlined below, modified from Chiou et al. [14]. Purified recombinant plasmid DNA (2.5 μg) was isolated using the Midi Plus plasmid DNA extraction system (Viogene, Taipei, Taiwan) and coated onto gold particles (1 μm diameter) for bombardment transformation. *Onc*. GR flower lips and leaves were incubated on sucrose-free 1/2 MS [43] solid medium and bombarded using a pneumatic particle gun (Biolistic PDS-1000/He; Bio-Rad) set to the following conditions: 1350 psi helium pressure of projectile; 27 mm Hg partial vacuum; 9 cm target-distance. Bombarded lips were subsequently incubated on MS solid medium at 22°C overnight for further experiments.

### Subcellular localization

Full-length cDNAs were amplified using PCR incorporating *Onc*. GR flower cDNA as template. The primer information is listed in Table 4. Products were cloned into pDONR221 by Gateway BP Clonase II Enzyme Mix (Invitrogen), and into p2YGWF (cGFP) and p2GWF7 (N-YFP) using Gateway LR Clonase II Enzyme Mix (Invitrogen) [44]. The plasmids were isolated and transformed into the lips using bombardment transformation. The transformed lips were observed on Zeiss LSM 510 META laser-scanning confocal microscope using an LD C-Apochromat 40×/1.1 W objective lens. Excitation wavelengths and emission filters were 458 nm/band-pass 465-510 nm for CFP, 488 nm/bandpass 500-530 nm for GFP, 514 nm/bandpass 525-555 nm for YFP, 561 nm/bandpass 575-630 nm for RFP and mCherry, and 488 nm/band-pass 650-710 nm for chloroplast autofluorescence. The images are presented as 3D maxima intensity projected stacks processed with LSM 510 version 4.2 (Zeiss).

**Table 4:** Primers used for the construction of fluorescence protein fusion

Gene	Primer No.	Sequence
**GFP**		
*OnTI1*	1894	AAAAAGCAGGCTTCATGGAGTTCATCAAAGCAAAG
	2129	AGA AAGCTGGGTCAGGAGTACACTTTTTAT
**YFP**		
*OnTI1*	1894	AAAAAGCAGGCTTCATGGAGTTCATCAAAGCAAAG
	1895	AGAAAGCTGGGTCTTAAGGAGTACACTTTTTATC
*OnTI2*	1892	AAAAAGCAGGCTTCATGGAGCTCATCAAATCATCA
	1893	AGAAAGCTGGGTCTTAATTACTAGTACATTTATT
*OnTI3*	2124	AAAAAGCAGGCTTCATGGAGTTCATCAAA
	2125	AGAAAGCTGGGTCTTAAGGAGTACACTGTT
*OnExpansin*	1898	AAAAAGCAGGCTTCATGACACCATCCCTCTTCCTC
	1899	AGAAAGCTGGGTCTCAAAACTGCGCGCCCTCGAA
*OnDRRP*	1896	AAAAAGCAGGCTTCATGGCTTCCTTCTCCTTCTCT
	1897	AGAAAGCTGGGTCCTAATTGTTGTTAAAGACAAC

### Promoter study

Promoters were amplified using PCR incorporating *Onc*. GR genomic DNA as the template. Primer information is given in Table 5. Using Gateway Technology (Invitrogen), products were cloned into pDONR221 using Gateway BP Clonase II Enzyme Mix (Invitrogen) and cloned into the binary vector, pHGWFS7 vector, which has a GFP-GUS reporter gene [44], using Gateway LR Clonase II Enzyme Mix (Invitrogen). These plasmids were co-transformed with a reference control, pJD301, containing the luciferase gene driven by the CaMV 35S promoter [45] into the *Onc*. GR lips and leaves using bombardment transformation. For histochemistry, bombarded tissues were transferred to a reagent containing 0.1 M phosphate buffer (pH 7.0), 0.5 mM potassium ferricyanide, 0.5 mM potassium ferrocyanide, 0.1% Triton X-100, 10 mM EDTA, 20% (v/v) methanol and 1 mM 5-bromo-3-indolyl-glucuronide (Sigma). The tissues were incubated overnight at 37°C and cleared using 70% (v/v) ethanol.

**Table 5:** Primers used in the promoter study.

Gene	Prom. size	Forward primer	Reverse primer
*OnTI1*	1913	AAAAAGCAGGCTGTCGACAAAGCCCAATTCATTCCAGT	AGAAAGCTGGGTCATCTAAAGTGATTGTGAGGA
	1340	AAAAAGCAGGCTTTCATGTTAACAACCATC	AGAAAGCTGGGTCATCTAAAGTGATTGTGAGGA
	920	AAAAAGCAGGCTCAACTTCATTTACTGTAGCTC	AGAAAGCTGGGTCATCTAAAGTGATTGTGAGGA
	740	AAAAAGCAGGCTTGAAAAATTGTGAG	AGAAAGCTGGGTCATCTAAAGTGATTGTGAGGA
	360	AAAAAGCAGGCTCGGAACTCCACAAG	AGAAAGCTGGGTCATCTAAAGTGATTGTGAGGA
*OnExpansin*	1027	AAAAAGCAGGCTGCCCCAAATGACACCTTA	AGAAAGCTGGGTCATTGTTAAGAGTTAGAATTTG
	882	AAAAAGCAGGCTCTCCTATTGCACCCATTTTC	AGAAAGCTGGGTCATTGTTAAGAGTTAGAATTTG
	746	AAAAAGCAGGCTTGATTCAACCCATTC	AGAAAGCTGGGTCATTGTTAAGAGTTAGAATTTG
	569	AAAAAGCAGGCTTGCACAGAGGCAAACATATATTT	AGAAAGCTGGGTCATTGTTAAGAGTTAGAATTTG
	334	AAAAAGCAGGCTCAACGCAAGTTAACC	AGAAAGCTGGGTCATTGTTAAGAGTTAGAATTTG
	133	AAAAAGCAGGCTCAGCATGTGCACTTCCACCT	AGAAAGCTGGGTCATTGTTAGAGTTAGAATTTG
*OnTI2*	1151	AAAAAGCAGGCTAAACAAGCTTCTCCCCCTTTGT	AGAAAGCTGGGTCATCTTAATAATTAGCTTGTTG
*OnDRRP*	1024	AAA AAG CAG GCT AGG AAG GAC ACA CAA CTT	AGA AAG CTG GGT CAT TAG AGA GTA GGA GGT

To measure luciferase and GUS activities, 0.4 g of tissue was ground in a mortar after liquid nitrogen treatment. A volume of 1 ml of 1× CCLR Buffer (Promega, Madison, WI, USA) was added to the powder and incubated at room temperature for 5 min. The solution was centrifuged at 18,000 × g for 5 min and the supernatant collected for further measurements. Luciferase activity was determined using luciferase assay reagent (Promega). GUS-specific activities were determined using 2 mM of 4-methylumbelliferone glucoronide substrate [14].

Transcription binding sites and tandem repeats were analyzed using the Plant Promoter Analysis Navigator [46].

## Authors' contributions

CTH carried out the majority of the experiments, including plasmid constructions and transient transformations. DCL performed the BAC library screening experiments. FHW, NTL, SJC, and SYT carried out the microarray experiments. SCS participated in the microscopy experiments. CHY participated in the *Oncidium *MADS gene studies. MTC and CSL designed and coordinated experiments. CSL wrote the manuscript. All the authors read and approved the final manuscript.
